# Metabolism, a Blossoming Target for Small-Molecule Anticancer Drugs

**DOI:** 10.3390/molecules30173457

**Published:** 2025-08-22

**Authors:** Michela Puxeddu, Romano Silvestri, Giuseppe La Regina

**Affiliations:** Laboratory Affiliated to Istituto Pasteur Italia-Fondazione Cenci Bolognetti, Department of Drug Chemistry and Technologies, Sapienza University of Rome, Piazzale Aldo Moro 5, 00185 Rome, Italy; michela.puxeddu@uniroma1.it (M.P.); romano.silvestri@uniroma1.it (R.S.)

**Keywords:** metabolism, cancer, aerobic glycolysis, glutamine metabolism, fatty acid synthesis

## Abstract

Reprogramming is recognized as a promising target in cancer therapy. It is well known that the altered metabolism in cancer cells, in particular malignancies, are characterized by increased aerobic glycolysis (Warburg effect) which promotes rapid proliferation. The effort to design compounds able to modulate these hallmarks of cancer are gaining increasing attention in drug discovery. In this context, the present review explores recent progress in the development of small molecule inhibitors of key metabolic pathways, such as glycolysis, glutamine metabolism and fatty acid synthesis. In particular, different mechanisms of action of these compounds are analyzed, which can target distinct enzymes, including LDH, HK2, PKM2, GLS and FASN. The findings underscore the relevance of metabolism-based strategies in developing next-generation anticancer agents with potential for improved efficacy and reduced systemic toxicity.

## 1. Introduction

**Figure 1:**
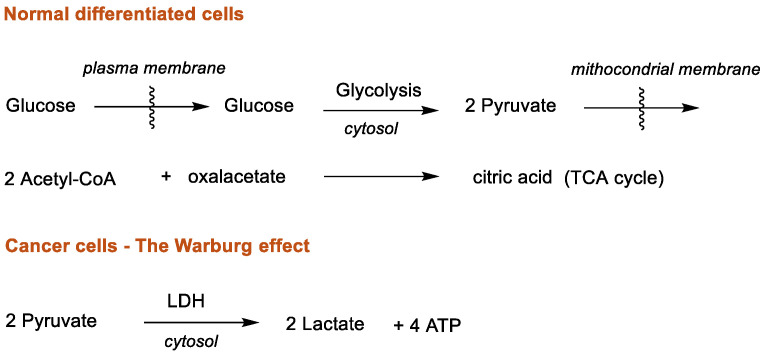
Aerobic glycolysis in normal and cancer cells.

The cancer metabolism was first suggested by the Nobel Prize Otto Warburg who observed that cancer cells produce additional energy in vitro by converting a larger amount of glucose into lactate compared to healthy cells [1]. This event, known as the Warburg effect, is referred to as aerobic glycolysis. The abnormal aerobic glycolysis promotes cell differentiation and cancer cell proliferation [2]. Under normal conditions, differentiated cells metabolize glucose to yield two molecules of pyruvate, two molecules of adenosine triphosphate (ATP) and two molecules of reduced nicotinamide adenine dinucleotide (NADH) (Figure 1). The two pyruvates are then converted into acetyl CoA, which enters the mitochondria and combines with oxaloacetate in the tricarboxylic acid (TCA) cycle to form citric acid [3]. Normally, one molecule of glucose produces about 30–34 molecules of ATP in the presence of oxygen [4]. In low-oxygen conditions or in the absence of oxygen, glucose is metabolized through anaerobic respiration. However, even in the presence of oxygen, cancer cells tend to convert pyruvate into lactate by lactate dehydrogenase (LDH) after glycolysis, producing four molecules of ATP [5,6]. Although this conversion is less efficient in terms of ATP yield compared to mitochondrial oxidation, it is faster [7] and continuously provides the cancer cell with amino acid, lipid, and nucleotide building blocks required for tumor proliferation [8,9,10]. As a consequence, a smaller proportion of pyruvate is converted into acetyl CoA prior to entering the TCA cycle, resulting in reduced production of NADH, FADH2 and ATP (Figure 1) [11].

## 2. Developing Therapeutic Agents Based on Metabolism

Targeting metabolism in medicinal chemistry holds significant potential for developing effective agents with highly specific mechanisms of action at catalytic and allosteric sites within hydrophobic pockets of metabolic enzymes. The success achieved by metabolism-targeting chemotherapy has demonstrated the effectiveness of this strategy for tumor treatment. The hallmarks of cancer include acquired capabilities, such as sustaining proliferative signaling, evading growth suppressors, enabling replicative immortality, inducing angiogenesis, and activating invasion and metastasis [12]. Deregulated cellular metabolism can be considered a core hallmark of cancer [13]. In addition to the Warburg effect, relevant reprogramming events that promote cancer cell survival and growth include de novo biosynthesis of proteins, nucleic acids and lipids. The anabolic metabolism of cancer cells includes increased lipid synthesis during proliferation to face the elevated demand for energy and for the formation of biological membranes [14,15]. The growing interest in compounds that interfere with cancer metabolism has been well documented in recent excellent reviews, such as that by Montesdeoca on lipogenic enzymes inhibitors [16], Stine on precision oncology [17] and Masci on colorectal cancer therapy [18]. Herein, we present the most recent advances in small molecules targeting metabolism, focusing on mechanism of action and their potential as antitumor drugs.

### 2.1. Aerobic Glycolysis

#### 2.1.1. Lactate Dehydrogenase (LDH)

**Figure 2:**
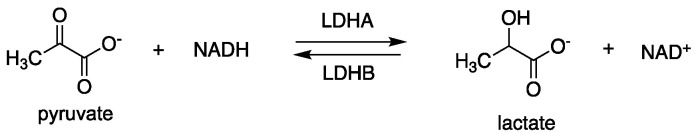
The general reaction catalyzed by LDH.

The release of the LDH in the tumor microenvironment promotes cancer cell proliferation and increases the likelihood of metastasis [19,20]. Inhibition of LDH by decreasing lactate release in the tumor microenvironment produces an useful anticancer effect [21]. Consequently, the search for LDH inhibitors as potential anticancer agents has become a topic of considerable interest [22,23,24]. LDH exists in five major isoforms, resulting from combinations of M and H monomers forming homo- and hetero-tetramers. The 4M and 4H tetramers correspond to LDHA and LDHB, respectively. LDHA catalyzes the conversion of pyruvate to lactate, whereas LDHB drives the reverse conversion (Figure 2) [25,26].

LDHA is induced by hypoxia or MYC activation, enabling glycolysis to become the primary source of ATP production in response to oxygen deprivation and the tumor’s ability to recruit new blood vessels [27]. As such, several research projects have focused on the discovery of natural [28] and small-molecule [29] LDH inhibitors.

**Chart 1:**
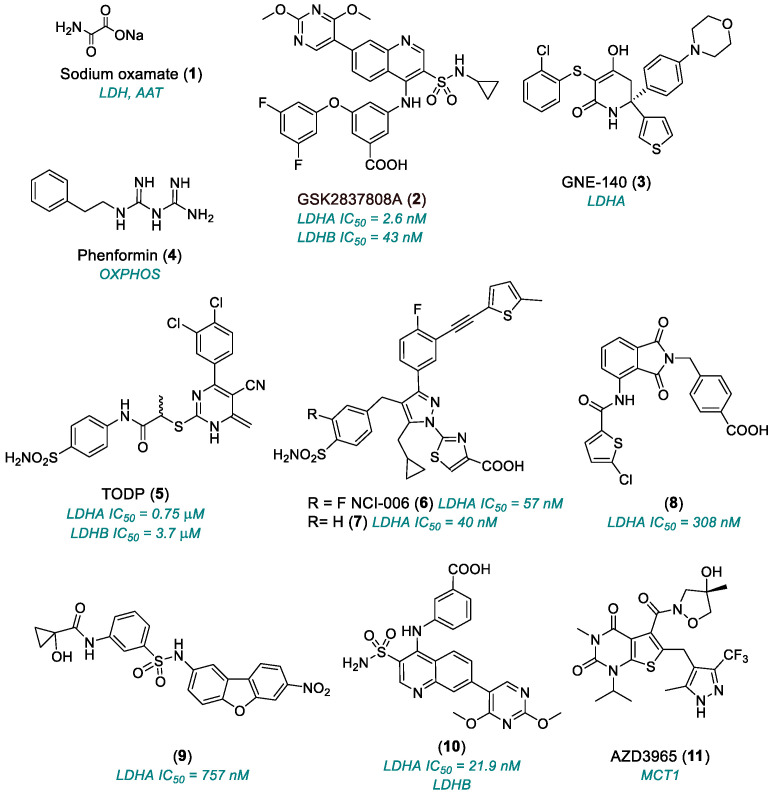
Chemical structures of LDH inhibitors **1**–**11.**

Sodium oxamate (**1**, Chart 1, Table 1) inhibits LDH and aspartate aminotransferase (AAT) and competes in vitro with pyruvate for the human LDHA. Oxamate and the AAT inhibitor amino oxyacetate suppressed proliferation of MDA-MB-231 cells and decreased tumor growth in MDA-MB-231 breast cancer xenografts in mice lacking the normal thymus gland [30]. Tumor suppressor p53 expression was strongly upregulated by (**1**), but only in glucose-treated cells, suggesting a connection with glycolysis inhibition [31]. While useful as a pharmacological tool, compound **1** shows limited enzymatic selectivity, weak potency and poor cellular uptake restricting its clinical utilization [32].

A series of quinoline 3-sulfonamides was found to inhibit potently and selectively LDHA and to rapidly reduce lactate production. GSK2837808A (**2**) inhibited LDHA at low nanomolar concentrations (IC_50_ = 2.6 ± 1.9 nM) and showed 80-fold selectivity over LDHB (IC_50_ = 43 ± 14 nM) in hepatocellular carcinoma cell lines [33]. LDHA inhibition by (**2**) suppressed glycolytic activity in temporomandibular joint osteoarthritis (TMJOA) synovial fluid (SF) containing >1000 kDa hyaluronic acid (HA). In the TMJOA microenvironment, vascularization and extracellular matrix (ECM) degradation correlate with increased nutrient consumption and active synovial proliferation [34]. As a result of LDHA inhibition by (**2**), HA production increased, and inflammation was reduced along with suppression of AMPK phosphorylation [35]. In Snu398 cells, which exhibit elevated glycolysis and pentose phosphate pathway activity, compound 2 reduced proliferation and induced strong changes in cell metabolism [33]. Pyruvate kinase (PK) activity increased in Snu398 but not in HepG2 cells, where the mitochondrial metabolism is significantly higher. Compound **2** also induced, in a dose-dependent manner, the inactive monomer of pyruvate kinase (PKM2) to form the M2 isoform, which catalyzes the final and rate-limiting reaction in the glycolytic pathway [36].

The 1,3-dihydropyrimidine GNE-140 (**3**) LDHA inhibitor rapidly affected the metabolism in MIA PaCa-2 human pancreatic cells, although cell death occurred only after two days of treatment [37]. Pancreatic cell lines relying primarily on oxidative phosphorylation (OXPHOS) for ATP production [38] were resistant to **3**, due to activation of the AMPK-mTOR-S6K signaling pathway, which promoted OXPHOS; the OXPHOS inhibitor phenformin (**4**) could resensitize these cells to **3**. Histone lysine lactylation (HKla) is a novel post-translational modification that connects cellular metabolism with epigenetic regulation. Although its role in the development of cardiac hypertrophy is still unclear, LDH inhibitor **3**, as well as **1**, has been shown to reduce HKla levels and mitigate cardiac hypertrophy development [39]. Compound **3** has also been shown to suppress osteogenic differentiation, proliferation, and migration while promoting apoptosis in alveolar bone marrow mesenchymal cells (ABMMCs). Mechanistically, these regulatory effects of lactate were thought to be mediated by HKla [40]. Compound **3** blocked dimethylsulfoxide-induced expression of Arg1, an M2 macrophage polarization marker, by lactate-H3K18la pathway in a methicillin-resistant Staphylococcus aureus (MRSA) disease model [41].

A screening of 2-thio-6-oxo-1,6-dihydropyrimidine derivatives led to the identification of a moderately potent LDHA inhibitor with an IC_50_ of 8.8 μM. Surface plasmon resonance (SPS) experiments indicated that simultaneous association with the NADH co-factor was required for an optimal binding to the LDHA. Based on these findings, medicinal chemistry work was conducted that aimed to improve the compound’s LDHA inhibition properties. As a result, compound TODP (**5**) inhibited LDHA with IC_50_ = 0.75 μM and LDHB with IC_50_ = 3.7 μM. A crystal structure of compound **5** in complex with LDHA at 1.90 Å resolution showed binding to Arg168, His192 and Asp165 residues of the LDH catalytic site and only the R-Me enantiomer was observed in the co-crystal structure [42].

Pyrazole-based LDH inhibitors were identified by quantitative high-throughput screening (qHTS) and structure-based design [42,43]. A lead compound effectively inhibited highly glycolytic MIA PaCa-2 (human pancreatic cancer) and A673 (human Ewing’s sarcoma) cell lines and showed favorable in vitro ADME properties although its PK profile was not suitable for in vivo use [44]. Optimization of the pyrazole-based chemotype yielded compounds **6** (NCI-006 NCATS-SM1440) and **7** (NCATS-SM1441), which stabilized LDHA in cells (IC_50_ 57 nM and 40 nM, respectively). Increasing the concentrations of compounds **6** and **7** resulted in NAD^+^ depletion and full glycolytic pathway inhibition, as evidenced by decreased extracellular acidification rate (ECAR). Both compounds **6** and **7** showed excellent metabolic profiles in hepatocytes and high plasma protein binding in mouse and human plasma. Structure–activity relationship (SAR) studies highlighted that the 5-methylthienyl group in the alkyne region is required for robust in vivo LDH inhibition [44,45]. Compound **6** and its analog NCI-737 (structure unavailable) were evaluated in in vitro and in vivo preclinical models of Ewing sarcoma (EWS), a highly metastatic bone cancer, constituting 10% to 15% of all bone sarcomas [46], predominantly afflicting adolescents [47]. Both compounds suppressed the glycolytic flux by impairing the conversion of pyruvate to lactate, disrupted the NAD^+^/NADH ratio and decreased the extracellular acidification rate (ECAR) in multiple EWS cell lines. Intravenous administration led to intratumoral drug accumulation, induced cell death and reduced tumor growth [48].

New phthalimide- and dibenzofuran-based selective LDHA allosteric inhibitors with sub-micromolar in vitro activity were identified. Co-crystal structures demonstrated that compounds **8** and **9**, with IC_50_ values of 308 nm and 757 nm, respectively, bound to a novel binding pocket distant from the polar, extended orthosteric substrate/cofactor site, while compound **10** (IC_50_ = 21.9 nm) was shown to hinder cofactor binding in an NADH competitive mode. Since few LDHA crystal structures show an allosteric pocket in an open conformation, the authors speculated that enzyme inhibition by (**8**) and (**9**) may depend on ligand-dependent conformational changes. Compound **10** also inhibited the LDHB, due to the high similarity among LDH isoforms active sites [49]. Allosteric inhibitors may take advantage by avoiding competition with endogenous substrates [50]. The architecture of the LDH protein plays a key role in enzymatic catalysis by tuning vibrational modes that promote chemical reactions [51]. Therefore, LDHA inhibition may result from conformational changes and/or dynamic processes crucial for enzymatic turnover [49].

AZD3965 (**11**) is a potent and selective inhibitor of monocarboxylate transporter 1 (MCT1), encoded by SLC16A1 [52], which is involved in lactate efflux following MYC-induced glycolysis [53]. Compound **11** potently inhibited MCT1 and MCT2, while sparing MCT3 and MCT4. It showed GI_50_ <100 nM (MTS growth assay) in diffuse large B-cell lymphoma (DLBCL) and non-Hodgkin lymphomas (NHL) cell lines. Inhibition of MCT1 led to decreased lactate efflux by (**11**). In in vivo studies, compound **11** was combined with doxorubicin or rituximab in DLBCL and Burkitt’s lymphoma models. Finally, when co-administered with a glutaminase (GLS) inhibitor, compound **11** enhanced cell death and growth inhibition compared to monotherapy treatment [53]. In a xenograft model of Raji lymphoblast-like cell in immunodeficient mice, compound **11** inhibited tumor growth, downregulated choline kinase alpha (which converts choline + ATP into phosphocholine + ADP + H^+^), and altered mRNA expression. These effects correlated with intracellular lactate accumulation induced by (**11**) [54]. In a multicenter phase I clinical trial, compound **11** was well tolerated at doses effective against MCT1-expressing cancers. Dose-limiting toxicities were mostly reversible, asymptomatic ocular changes. A dose of 10 mg twice daily was recommended for phase II trials [55].

#### 2.1.2. Glucose Transporter 1 (GLUT1)

**Figure 3:**
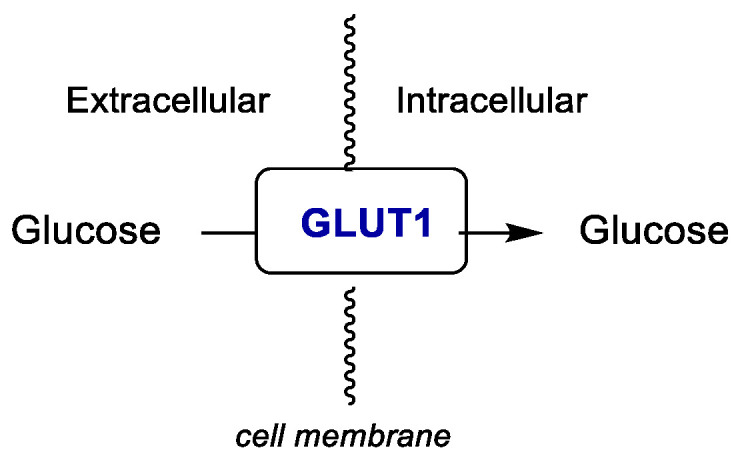
GLUT1 function.

Glucose transporter type 1 (GLUT1) is the main transporter responsible for glucose uptake in many tissues (Figure 3). It is expressed in most tissues, but is highly abundant in brain endothelial cells, glial cells, erythrocytes and placenta [56]. GLUT1 is a glycoprotein belonging to the GLUT family of carriers and is responsible for the basal glucose uptake across the blood–tissues barriers, including the blood–brain barrier (BBB) [57]. Alterations in GLUT1 impair the glucose supply to glial cells and neurons. A reduction in brain glucose transport, primarily caused by mutations in the SLC2A1 gene encoding GLUT1 [58], is the etiological basis of the glucose transporter type 1 deficiency syndrome (GLUT1DS), an autosomal dominant disorder [59,60]. The classical form of GLUT1DS presents as early-onset encephalopathy (during the first year of life), characterized by severe epilepsy, a complex movement disorder and developmental delay, including microcephaly, due to immature tight junctions in the BBB that allow paracellular glucose transport [61].

**Chart 2:**
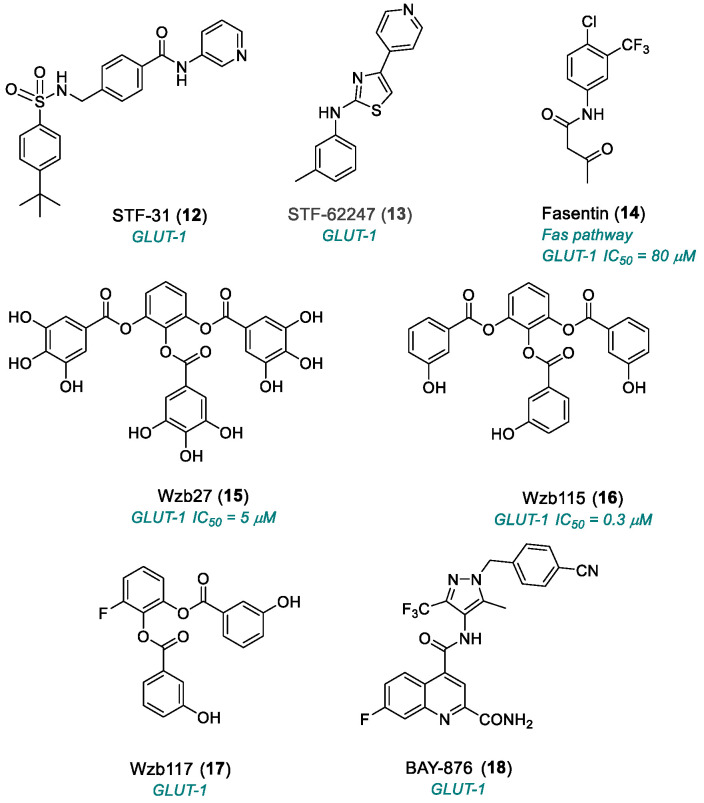
Chemical structures of GLUT1 inhibitors **12**–**18.**

Approximately 80% of renal cell carcinomas (RCCs), similar to many other cancers, are associated with the loss of the von Hippel–Lindau (VHL) tumor suppressor gene, which is correlated with the Warburg effect and partially results from GLUT1 upregulation. Compound STF-31 (**12**) (Chart 2) was identified through a high-throughput screening of ~64,000 small molecules, aimed at discovering agents with selective lethality in VHL-deficient RCC cells. Two classes of compounds emerged from this screening: sulfonamides and pyridyl anilino thiazoles, including compound **12** and STF-62247 (**13**), respectively. RCC4 cells lacking VHL showed reduced viability in the presence of (**12**) in a dose-dependent manner compared to their wild-type cells RCC4/VHL. The clonogenic assay confirmed that compound **12** selectively targeted RCC4 cells. It did not induce any morphological or biochemical features of autophagy, nor did it deregulate HIF expression in VHL-deficient cells. Importantly, lactate production and extracellular acidification were significantly inhibited. Compound **12** decreased glycolysis by inhibiting glucose transport rather than targeting a particular glycolytic enzyme. Moreover, it was toxic to RCCs expressing GLUT1 but low or undetectable levels of GLUT2, indicating that STF-31 acts as a high-affinity GLUT1 inhibitor [62].

STF-62247 (**13**) was identified by screening for small molecules targeting selectively VHL-deficient RCC cells. Compound **13** induced cytotoxicity and reduced tumor growth in VHL-deficient RCC cells compared to VHL wild-type (WT) cells. The cytotoxicity of (**13**) was mediated through autophagy in a HIF-independent mechanism [63]. SAR studies identified several key features necessary for selective cytotoxicity against VHL-negative RCC cells: the aniline-NH and the 4-pyridyl-N groups likely participate in H-bonding, while an unsubstituted thiazole is required for VHL selectivity. Small lipophilic substituents at the 3- and 4-positions of the aniline ring suggested the presence of a lipophilic pocket within the target protein [64].

Fasentin (**14**) is a small molecule that sensitizes the Fas-signaling pathway (central to programmed cell death) by effecting glucose uptake. At 80 μM, (**14**) dramatically reduced glucose uptake in leukemia and prostate cancer cells [65]. Compound **14** was also used to shed light on the role played by GLUT1 in corticotropin-releasing hormone (CRH)-mediated glucose uptake in adenomatous corticotropes [66]. These findings have prompted the development of anticancer strategies based on glucose metabolism. Compound **14** inhibited endothelial cells growth without compromising their survival and reduced the number of enterochromaffin (EC) cells, tumor cells and, to a lesser extent, fibroblasts. At 50 nM, it impaired angiogenesis in the chick chorioallantoic membrane (CAM) assay. Notably, this anti-angiogenic activity was independent of glucose metabolism modulation. In human dermal microvascular endothelial cells, (**14**) reduced the uptake of glucose. It is worthy to note that **13** failed to inhibit tube formation in these cells [67].

Compounds Wzb27 (**15**) and Wzb115 (**16**) (IC_50_ = 5 μM and 0.3 μM, respectively) were serendipitously identified as inhibitors of basal glucose transport [68] during a screening for molecules that stimulate insulin receptor-mediated glucose uptake [69]. These compounds proved more potent than other GLUT1-inhibitory agents, such as (**14**) or apigenin, a natural product with anti-inflammatory and antitumor activity, in inhibiting basal glucose transport and cancer cell proliferation. Both (**15**) and (**16**) induced G1/S cell cycle arrest and apoptosis in lung and breast cancer cells without significantly affecting their normal cell counterparts. Apoptosis was mediated by caspase-3 activation, independent of p53, consistent with a glucose deprivation mechanism [68]. The analog compound WZB117 (**17**) inhibited cancer cell proliferation both in vitro and in vivo in a nude mouse model. It proved to inhibit glucose transport in human RBCs having GLUT1 as the only transporter. Compound **17** reduced GLUT1 protein levels, intracellular ATP, and glycolytic enzymes, while increasing AMP-activated protein kinase (AMPK). ATP depletion and induction of senescence were observed in GLUT1 inhibitor-treated cancer cells [70]. Compound **17** reversibly and competitively inhibited 3-O methylglucose (3MG) uptake in human erythrocytes but acted as a non-competitive inhibitor of glucose efflux. In HEK293 cells, (**17**) inhibited Glut4 more potently than GLUT1 and GLUT3 [71].

A high-throughput screening campaign was led to identify *N*-(1H-pyrazol-4-yl)quinoline-4-carboxamides as a promising scaffold for the development of GLUT1-selective small-molecule inhibitors. SAR studies led to single-digit nanomolar inhibitors with selectivity over GLUT2, GLUT3 and GLUT4. The most promising compound, BAY-876 (**18**), showed high GLUT1 potency and selectivity thanks to the presence of an unsubstituted amide at position 2 and a fluorine atom at position 7, respectively, of the quinoline ring. Compound **18** exhibited good in vitro metabolic stability and high oral bioavailability in vivo, with low clearance in rats and dogs [72]. Inhibition of GLUT1 by (**18**) halted glucose uptake in cancer cells, induced apoptosis, and suppressed the TNF-α-induced interleukin-8 (IL-8) production in head and neck squamous cell carcinoma (HNSCC) cells. BAY-876 reduced the viability of SCC47, RPMI2650, and FaDu cells after 24 h and induced apoptosis at higher concentrations in some cancers. These results highlight GLUT1 as a promising target in HNSCC of the upper aerodigestive tract [73].

#### 2.1.3. Hexokinase (HK)

**Figure 4:**
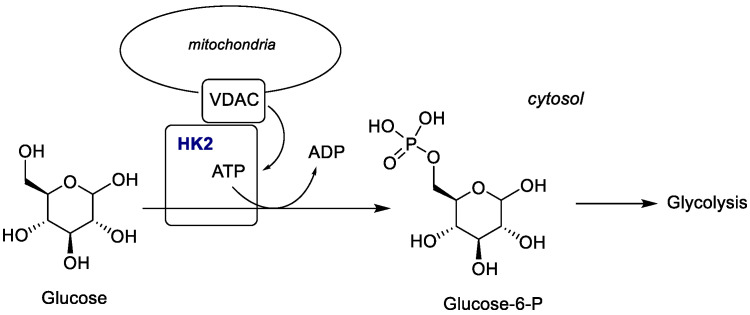
Hexokinase function in glucose metabolism.

HK catalyzes the conversion of glucose to glucose-6-phosphate (G6P), the first irreversible step of glycolysis, and is involved in pathways associated with cancer cell growth, such as nucleotide and lipid synthesis, tricarboxylic acid cycle and pentose phosphate [74]. In humans, five HK isoenzymes are recognized: HK1, HK2, HK3, HK4 and the HK domain-containing protein 1 (HKDC1) [75]. Among them, HK2 isoenzyme was found to be overexpressed in various cancer types [76,77], while HK1 is the predominant isoform in normal cells. Due to its key role in reprogrammed glucose metabolism, HK2 has emerged as an attractive target for anticancer drug discovery [78]. The transport of mitochondrial metabolites across the outer mitochondrial membrane (OMM) is mediated by voltage-dependent anion channels (VDACs) [79] (Figure 4), with VDAC1 being the most prominent isoform, frequently overexpressed in several types of cancer [80]. HK2 was found to be associated with VDAC1 at the OMM, allowing ATP transport from mitochondria to the cytosol as ADP to convert glucose into glucose-6-phosphate. Moreover, HK2 contributes to apoptosis evasion in cancer cells by binding to VDAC1 and competing with Bax [81]. Thus, inhibition of the HK2-VDAC1 interaction turned out to be a promising strategy for cancer therapy. The crystal structure of HK2 (PDB ID: 2NZT, resolution: 2.45 Å) was used to design HK2 inhibitors. Additional inhibitors were developed to target the G6P-binding site by mimicking the G6P interaction.

Due to the high sequence similarity between rat HK1 (PDB ID: 1BG3, resolution: 2.80 Å) and human HK2, a homology model was constructed to study how VDAC1 phosphorylation may disrupt HK2 binding. For docking studies to HK2, the structure of VDAC1 (PDB ID: 2JK4, resolution: 4.10 Å) was used [78].

**Chart 3:**
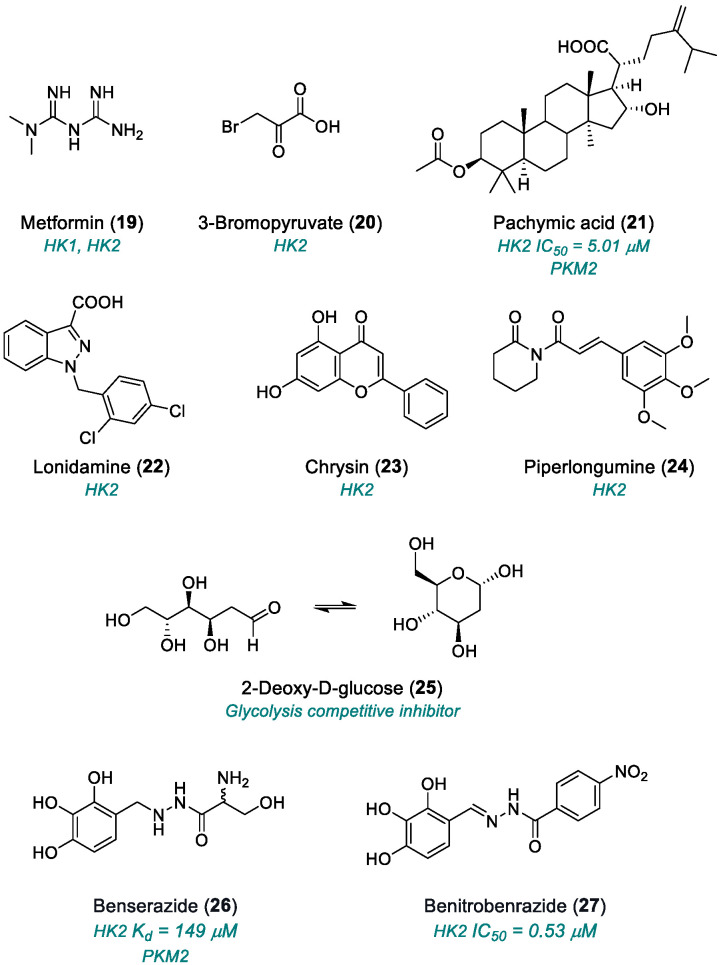
Chemical structures of HK2-VDAC1 inhibitors **19**–**24** and glucose binding site inhibitors **25**–**27.**

HK2-VDAC1 inhibitors. Metformin (**19,**Chart 3) was shown to impair energy balance in cancer cells in vitro by the selective enzymatic inhibition of HK1 and HK2 isoforms [82]. This activity was demonstrated in MDA-MB-231 triple-negative breast cancer cells in xenograft models [83]. Both isoforms were localized to the mitochondrial outer membrane; HK2′s membrane association was found to be partially dependent on G6P [84]. HK2 inhibition restricts cancer cell growth by limiting ATP access for glucose phosphorylation [85]. Notably, prolonged treatment with compound **19** caused a marked reduction in tumor growth without any visible necrosis, whereas acute drug administration resulted in profound cytotoxic effects [83].

3-Bromopyruvate (3-BrPA, **20**) covalently modifies the HK2 protein, leading to the release of the apoptosis-inducing factor (AIF) from the mitochondria to cytosol as a consequence of the interaction of HK2 with AIF. The dissociation of HK2 from mitochondria alone was sufficient to cause apoptosis, particularly in the mitochondria-deficient ρ^0^ cells with high HK2 expression, without affecting mitochondrial membrane potential, ROS generation, or oxidative phosphorylation [86].

Pachymic acid (**21**) is a triterpenoid compound obtained from *Poria cocos* (Polyporaceae), a saprophytic fungus that grows on various Pinus species [87]. Compound **21** has demonstrated anti-inflammatory, anticancer, anti-aging, and insulin-like properties, which directly inhibited the HK2 with an IC_50_ of 5.01 μM, inducing mitochondrial dysfunction, ATP depletion, and ROS generation. Compound **21** acts as a competitive activator of PKM2 by mimicking fructose-1,6-bisphosphate, the natural activator. It was suggested that this compound could be a G6P mimic [88].

Lonidamine (**22**) is an anticancer agent approved as a monotherapy for advanced breast, prostate, lung and brain cancers. Its mechanism of action involves selective inhibition of aerobic glycolysis in tumor cells [89] through HK inhibition [90] and disruption of the mitochondrial membrane [91]. Compound **22** was administered alone or in combination with other chemotherapeutic agents (i.e., paclitaxel, docetaxel, 5-fluorouracil and 2-desoxy-2-glucose), surgery or radiation therapy in a sustained-release formulation to treat cancer, inflammation and autoimmune disease [92].

Chrysin (**23**) is a flavonoid compound extracted from natural sources, such as blue passion flower, propolis and honey, commonly used in traditional Chinese medicine. It showed antitumor activity in several human cancer cell lines and also possesses antioxidant, anti-inflammatory and antibacterial properties. Its antitumor activity involves activation of the extrinsic apoptosis pathway [93], alteration of cyclin-dependent kinases (CDKs) [94] and interference with key signaling pathways, including Ras-Raf-MAPKs, PI3K-Akt, STAT, NF-κB, Wnt-β-catenin and Notch [95,96]. Compound **23** inhibited glycolysis and induced apoptosis in hepatocellular carcinoma (HCC) cells both in vitro and in vivo, through inhibition of HK-2 (which was found to be overexpressed in the majority of HCC tissue) and tumor glycolysis and activation of mitochondria-associated apoptosis [97].

Piperlongumine (**24**), a natural compound isolated from long pepper piper longum L, showed anticancer activity against several human tumor types, including lung, liver, prostate, breast and colorectal cancers. Compound **24** inhibited the non-small-cell lung cancer (NSCLC) both in vitro and in vivo by downregulating glycolysis, decreasing HK2 expression and inducing mitochondrial apoptosis. The glycolytic suppression induced by (**24**) was mediated by the Akt signaling pathway [98].

Glucose-binding site inhibitors. 2-Deoxy-D-glucose (2-DG) (**25**) is a glucose analog bearing a hydrogen atom replacing the hydroxyl group at position 2, functioning as a competitive inhibitor of glycolysis [99,100]. It inhibits several glycolytic enzymes, leading to cell death. Compound **25** fulfills Lipinski’s rule of five and shows activity against hypoxic tumor cells, while also reducing oxygen dependency, including in the context of COVID-19 [101]. Due to its structural similarity to D-mannose, (**25**) interferes with glycosylation processes and induces endoplasmic stress [102,103]. Compound **25** has shown potential as a cancer and antiviral agent and enhanced the efficacy of paclitaxel when co-administered or co-encapsulated in nanoparticles for a lung cancer model [104]. Compound **25** was also shown to suppress the inflammation in innate immune cells [105] and the expression of herpes simplex virus type-1 receptor [106] by modulating anti-inflammatory mediators and the polarization of macrophages [107]. Applications of (**25**) as an anticancer agent have been reported in combination with the Bcl-2 antagonist ABT-263 and ABT-737 (ABT) [108], or with other therapeutic agents or radiotherapy to overcome its limited therapeutic effect [109]. 2-Halogenated D-glucose analogs were found to be more potent than (**25**) in killing hypoxic tumor cells and may be more clinically effective when combined with standard chemotherapeutic protocols [110]. The combination of low-dose (**25**) and (**19**) synergically inhibited cyst formation and human polycystic kidney cell proliferation [111] and proved effective across a broad spectrum of preclinical cancer models [112].

Benserazide (**26**) was identified as an HK2 inhibitor (K_d_ of 149 ± 4.95 μM) by structure-based virtual screening studies on FDA-approved drugs and nutraceuticals from the ZINC Drug Database [113]. Due to its inhibitory effect on peripheral dopa decarboxylase, compound **26** is used as a levodopa coadjuvant in standard Parkinson’s disease treatment. It showed approximately 8-fold selectivity for HK2, versus HK4, and about 5-fold selectivity against HK2 over HK1. (**26**) inhibited SW480 cancer cells proliferation and CRC xenograft growth by targeting HK2, inducing apoptotic cell death through energetic stress and activation of AMPKα, p53 and p27, along with loss of mitochondrial membrane potential [114].

Benitrobenrazide (**27**) is a nanomolar inhibitor of HK2 (IC_50_ of 0.53 ± 0.13 μM), induces apoptosis and inhibits proliferation of HK2-overexpressed cancer cells. It significantly inhibited glycolysis and cancer cell proliferation both in vitro and in vivo with low toxicity by directly targeting HK2. Upon oral administration, compound **27** effectively suppressed tumor growth in SW1990 and SW480 xenograft models [115].

**Chart 4:**
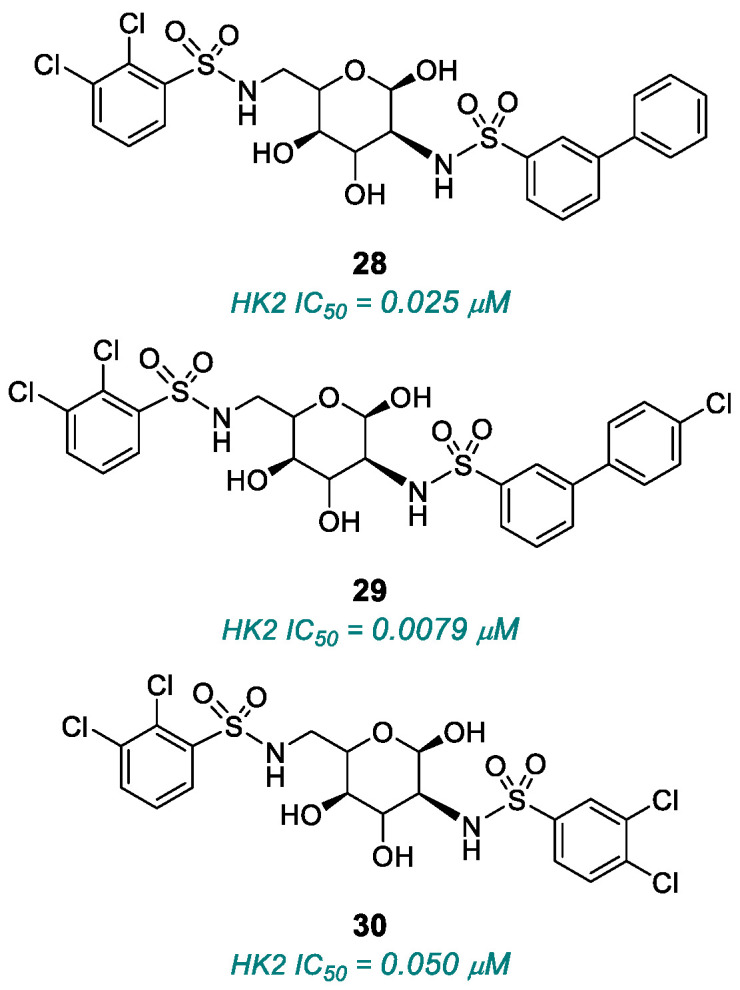
Chemical structures of glucose-6P-mimicking binding site **28**–**30.**

Glucose-6P-mimicking binding site inhibitors. Metformin (**19**) has also been reported to inhibit HK2 at μM concentrations similar to those of G6P (85). Glucose-6P binds to an allosteric site near, but distinct from, the glucose-binding site in the HK1 [116]. Co-crystallography studies revealed that the first structure of HK2 bound a ligand and G6P at 2.76 Å [117]. The co-crystal structure of HK2 highlighted the presence of a highly flexible binding site. SAR studies were conducted to enhance HK2 selectivity over HK1, given their high sequence similarity and conserved glucose-binding sites [118]. A 6-sulfonamide linker turned out to optimally mimic the phosphate group of G6P. Compounds **28**–**30** (Chart 4) were identified as potent and selective HK2 inhibitors, with IC_50_ values of 0.025, 0.0079 and 0.050 μM, respectively [117].

#### 2.1.4. Pyruvate Kinase (PK)

**Figure 5:**
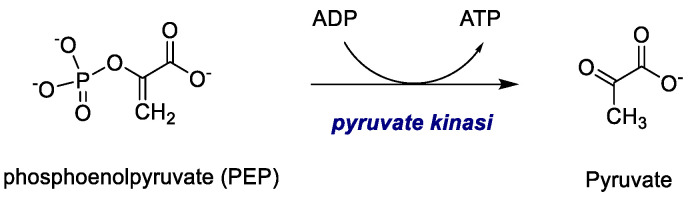
PK role in glycolysis.

Pyruvate kinase (PK) is a rate-limiting glycolytic enzyme that catalyzes the final step of glycolysis, converting phosphoenolpyruvate (PEP) into pyruvate [119] (Figure 5). Among the four reported PK isoforms, PKM1 and PKM2 are expressed in several cell types, including neutrophils, while PKL is mainly found in the liver and PKR in erythrocytes [120]. The enzymatic activity of PKM2 is tightly regulated by endogenous allosteric effectors and intracellular signaling pathways, which influence both its enzymatic activity and oligomeric state. PKM2 can assume a dimeric or monomeric form, leading to the accumulation of glycolytic intermediates. The dimeric form of PKM2 can translocate to the cell nucleus, where it co-activates transcription factors, regulating genes involved in cell proliferation and glycolysis. Upon allosteric activation, PKM2 adopts a tetrameric conformation with high glycolytic activity [121]. ROS production in neutrophils is dependent on glycolytic flux. PKM2 regulates the dihydroxyacetone phosphate (DHAP) and diacylglycerol (DAG) levels, which activate protein kinase C (PKC), and, in turn, the NADPH oxidase complex, thereby promoting ROS production and neutrophil cytotoxic activity [122]. PKM2 was identified as a promising target for cancer therapy, with subcellular localization of PKM2 proposed as a potential biomarker for therapeutic response in non-small-cell lung cancer (NSCLC) cell lines [123].

The previously cited compound **26** was also identified as a novel inhibitor targeting PKM2 for melanoma treatment [124]. It also showed cystathionine-β-synthase (CBS) inhibitory activity among 8871 clinically used drugs, with relative selectivity versus cystathionine-γ lyase (CSE) and 3-mercaptopyruvate sulfurtransferase (3-MST). Benserazide (**26**) inhibited colon cancer cell proliferation in HCT116 and HT29 cell lines, both highly expressing CBS, impaired cellular bioenergetics in HCT116 cells and suppressed colon cancer tumor growth in mice [125].

**Chart 5:**
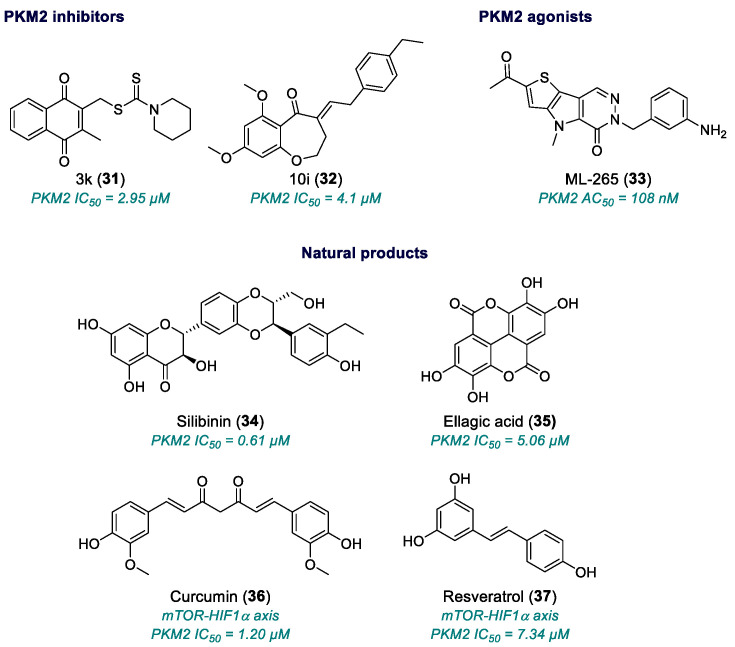
Chemical structures of PKM1 inhibitors and agonists **31**–**37.**

SAR studies on naphthoquinone derivatives led to the identification of thiocarbamate 3k (**31**, Chart 5) as a selective PKM2 inhibitor (IC_50_ = 2.95 ± 0.53 μM) with antiproliferative activity in PKM2-overexpressing HCT116, HeLa and H1299 cells, superior to the known PKM2 inhibitor shikonin [126]. Two novel 2,3-didithiocarbamate analogs of compound **31** exhibited even stronger inhibition of PKM2; their activities were superior to (**31**), showing dose-dependent cytotoxicity with IC_50_ values in nanomolar concentrations against HCT116, MCF7, HeLa, H1299 and B16 cells [127].

A series of benzoxepane derivatives, inspired by marine-derived natural products, was synthesized as novel anti-inflammatory agents. PKM2 was identified as the molecular target of compound 10i (**32**) (IC_50_ = 4.1 μm) [128]. Compound **32** exhibited anti-inflammatory activity in lipopolysaccharides (LPS)-stimulated RAW264.7 macrophages by inhibiting TNF-a protein release. In mouse primary microglia 10 μM of (**32**) showed potent anti-neuroinflammatory effects, with an inhibition rate of 87.9% and low toxicity. It inhibited PKM2 kinase activity without affecting PKM2 protein expression in vitro in a cell-free assay. After incubation in both RAW264.7 and mouse primary microglia, compound **32** caused a significant reduction in LDHA and NOD-like receptor protein 3 (NLRP3) inflammasome activation, consistent with the role of PKM2 in promoting glycolysis through transcriptional regulation of glycolysis-related genes [129,130].

ML-265 (**33**) was identified to study the neuroprotective effects of pharmacologically reprogramming photoreceptor metabolism by altering PKM2 function (AC_50_ = 108 ± 20 nM) during outer retinal apoptotic stress [131]. Compound **33** increased pyruvate kinase activity in 661W cells, a retinal ganglion precursor-like cell line in which glaucoma-associated optineurin mutants selectively induce cell death [132], both in vitro and in vivo following intravitreal injection in rat eyes, without affecting the expression of glucose metabolism-related genes [131]. After showing good microsomal stability, aqueous solubility and CaCO_2_ permeability, compound **33** was progressed to in vivo PK, where it showed good oral bioavailability, sustained exposure, long half-life and low clearance. In a 7-week mouse xenograft model, ML265 significantly reduced tumor size and occurrence without showing signs of acute toxicity [133].

Natural products. Silibinin (**34**) and ellagic acid (**35**) showed competitive inhibition of PKM2, with Ki values of 0.61 μM and 5.06 μM, respectively, while curcumin (**36**) and resveratrol (**37**) acted as non-competitive inhibitors of PKM2, with Ki values of 1.20 μM and 7.34 μM, respectively [134].

Silibinin (**34**) is the most bioactive component of silymarin, an extract obtained from milk thistle (Silybum marianum L. Gaertn.) [135]. Silymarin has demonstrated anticancer effects against several tumors, including lung, prostate, colon, breast, bladder and hepatocellular carcinoma [136]. Treatment with 75–100 μM of (**34**) for 24–48 inhibited histone deacetylases (HDAC) and DNA methyltransferase (DNMT) in H1299 human H1299 NSCLC cell lines [137]. At 300 μM, silibinin significantly inhibited DNMT activity in SW480 and SW620 colon cancer cells, with only weak HDAC inhibition [138]. Combined treatment with trichostatin A (TSA) and (**34**) demonstrated a synergistic effect on cancer cell death via DNMT inhibition [138]. Furthermore, compound **34** was shown to modulate non-coding RNAs (ncRNAs), which play a critical role in the epigenetic regulation of gene expression. In triple-negative breast cancer (TNBC) cells, (**34**) disrupted the metabolic reprogramming via modulation of the EGFR-MYC-TXNIP signaling pathway. Drug combination of compound **34** with paclitaxel demonstrated synergistic antiproliferative activity in TNBC models [139].

Ellagic acid (**35**) is a polyphenolic compound found in various plant-derived foods that was approved by the Japanese Ministry of Health, Labor and Welfare as an antioxidant supplement [140]. Beyond its free radical scavenging activity, (**35**) has gained interest as a tumor chemopreventive agent [141], also showing protective effects against chronic alcohol-induced liver damage in rats [142]. Its antitumor activity has been mostly correlated to direct inhibition of cell proliferation and induction of apoptosis. In small clinical studies, it has been evaluated in combination with standard chemotherapy in colorectal and prostate cancer patients [143]. Compound **35** showed high binding affinity to Cyclin-Dependent Kinase 6 (CDK6), downregulated its protein expression at the translational level [144] and inhibited cell growth in different human cancer cells lines [145]. In A549 cells, ellagic acid induced apoptosis via phosphoinositide 3-kinase/protein kinase B pathway in a CDK6-dependent manner [144].

Curcumin (**36**) is a plant-derived polyphenolic compound, found in turmeric (Curcuma longa), widely used in both traditional and modern medicine. It exhibits antioxidant, anti-inflammatory, antiviral, anti-angiogenic, anti-HIV-1 and anticancer activity due to its ability to adopt different conformations that facilitate interaction with different targets [146]. Compound **36** inhibited glucose uptake and lactate production in several cancer cell lines by downregulating PKM2 expression through the inhibition of the mTOR-HIF1α axis [147].

Resveratrol (**37**) (3,4′,5-trihydroxystilbene) is a phytoalexin found in the skin of red grapes and other fruits [148]. It exerts anticancer activity in all stages of tumor development: initiation, promotion and progression [149]. Compound **37** exerts a PKM2-mediated effect on cancer metabolism, decreasing PKM2 mRNA and protein expression via mTOR inhibition in HeLa, HepG2 and MCF-7 cells [150]. Since the mTOR pathway regulates PKM2 expression [151], PKM2 overexpression can reverse the effects of resveratrol. Several signaling pathways, involved in carcinogenesis and host defense response, are also modulated by resveratrol, accounting for its pleiotropic anticancer activity. In combination with 5-f or cisplatin, (**37**) displayed a synergistic effect and increased cancer cell sensitivity [152].

**Table 1:** Compounds targeting aerobic glycolysis.

Name	Target	Year	Stage	Limitation	References
Sodium oxamate (**1**)	LDHA, AAT	1965	Preclinical studies	Not suitable for clinical utilization	[30,31,32]
GSK2837808A (**2**)	LDHA	2013	Preclinical studies	Poor oral bioavailability; very rapid clearance in vivo; very low plasma concentrations	[33,34,35,36]
GNE-140 (**3**)	LDHA	2015	Preclinical studies	Unfavorable pharmacokinetics; rapid clearance	[37,38,39,40,41]
TODP (**5**)	LDHA	2013	Preclinical studies	Unfavorable pharmacokinetics	[42]
NCI-006 (**6**)	LDHA	2020	Advanced preclinical candidate	Low cell permeability; high plasma binding; potential target saturation	[45]
(**8**), (**9**)	LDHA	2020	Preclinical Studies	Only enzymatic assay	[49]
(**10**)	Allosteric site LDH	2020	Preclinical studies	Only enzymatic assay; not selective for LDHA (also active on LDHB)	[49]
AZD3965 (**11**)	MCT1/2	2017	Phase I (2020)	Reduced efficacy in glycolytic/resistant tumors; potential ocular and cardiac toxicities; limited to lymphomas; limited systemic pharmacology and long-term data	[52,53,54,55]
STF-31 (**12**)	GLUT1	2011	Preclinical studies	No PK data available; necrotic cell death; complex mechanism	[62]
STF-62247 (**13**)	GLUT1	2011	Preclinical studies	Undefined pharmacokinetics	[62,63]
Fasentin (**14**)	Fas pathway GLUT1	2006	Preclinical studies	Undefined pharmacokinetics	[66]
WZB27 (**15**)	GLUT1	2010	Preclinical studies	Moderate potency; no PK studies	[68,69]
WZB115 (**16**)	GLUT1	2010	Preclinical studies	Moderate potency; no PK studies	[68,69]
WZB117 (**17**)	GLUT1	2012	Preclinical studies	Limited selectivity; no PK studies; in vivo instability	[70,71]
BAY-876 (**18**)	GLUT1	2016	Preclinical studies	Limited cellular activity; effects on normal cells; tumor metabolic adaptation	[72]
Metformin (**19**)	HK1, HK2	2013	Preclinical studies	Cytotoxic effect	[82,83]
]3-BrPA (**20**)	HK2	2009	Preclinical studies	Systemic toxicity; non-selective reactivity; acidic pH dependence; metabolic adaptation;	[86]
]Pachymic acid (**21**)	HK2, PKM2	2015	Preclinical studies	Poor in vivo bioavailability; narrow therapeutic window; moderate potency; multiple and partly selective mechanisms; lack of systematic toxicity data	[87,88]
Lonidamine (**22**)	HK2, MCT	1981	Fase II–III (only in combination)	Mitochondrial toxicity; low oral bioavailability	[89,90,91,92]
Chrysin (**23**)	HK2, PI3K/Akt, NF-κB	2010	Preclinical studies	Poor oral bioavailability	[93,94,95,96,97]
]Piperlongumine (**24**)	PI3K/Akt/mTOR e HIF-1α	2011	Preclinical studies	Low bioavailability; poor metabolic stability	[98]
2-DG (**25**)	Glycolysis competitive inhibitor	2019	Phase II completed	Systemic toxicity; metabolic compensation	[99,100,101,102,103,104]
Benserazide (**26**)	HK2, PKM2	2017	Preclinical studies	Off-label repositioning; neurological effects	[113]
Benitrobenrazide (**27**)	HK2	2021	Advanced preclinical studies	Aggregation tendency; poor ADME/Tox characterization; activity on minor HK isoenzymes	[115]
(**28**), (**29**), (**30**)	HK2	2015	Preclinical studies	Micromolar potency; incomplete selectivity	[117]
3k (**31**)	PKM2	2017	Preclinical studies	Low potency; relative selectivity; no full pharmacological development	[126]
10i (**32**)	PKM2	2019	Preclinical studies	Neuroinflammatory/ischemic application only; no published antitumor validation	[128]
ML-265 (**33**)	PKM2	2012	Advanced Preclinical studies	Variable cell-based potency; poor solubility; need for optimized formulation; incomplete toxicological data	[131,132,133]
Silibinin (**34**)	PKM2	2003	Phase II	Effective only at micromolar range	[134,135,136,137,138,139]
Ellagic acid (**35**)	PKM2	1996	Small phase I/II studies (in combination)	Effective only at micromolar range	[140,141,142,143,144,145]
Curcumin (**36**)	mTOR-HIF1α axis	1990	Phase I/II completed, few phase III studies	Low bioavailability; high dosing; standardization issues	[146,147]
Resveratrol (**37**)	PI3K/Akt/mTOR, HIF1α	1997	Phase I/II	Very low bioavailability; rapid metabolism; high doses needed	[148,149,150,151,152]

### 2.2. Glutamine Metabolisms

**Figure 6:**
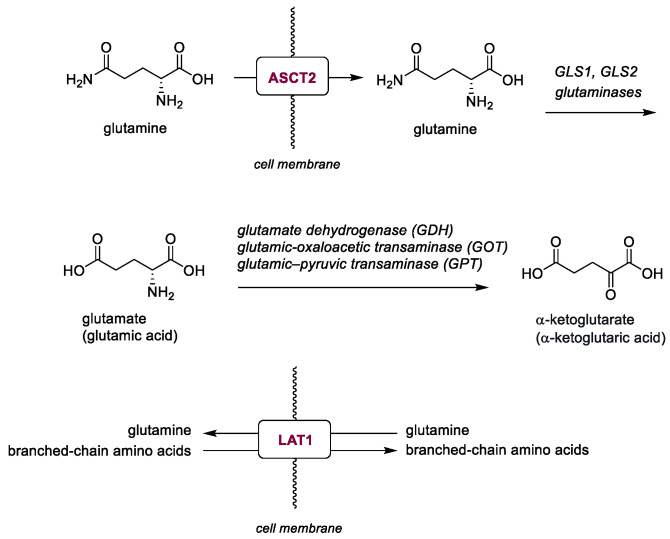
Glutamine metabolism.

Mitochondrial glutaminases GLS1 and GLS2 catalyze the deamination of glutamine to glutamate. Glutamate is then converted into α-ketoglutarate by glutamate dehydrogenase, glutamic-oxaloacetic transaminase (GOT) or glutamic–pyruvic transaminase (GPT), entering the TCA cycle (Figure 6).

**Chart 6:**
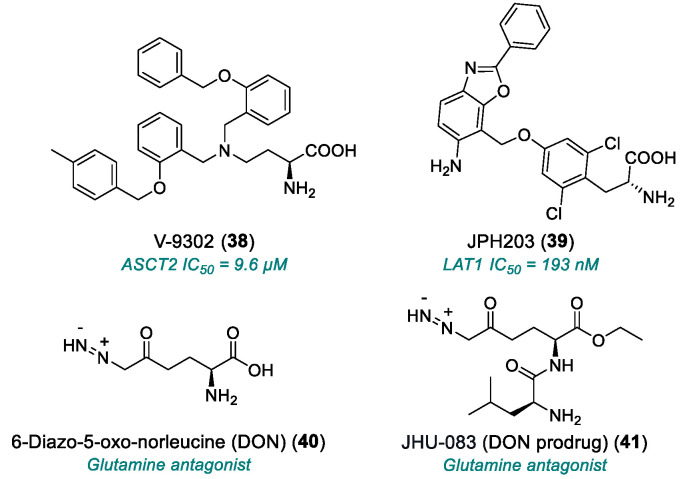
Chemical structures of glutamine uptake inhibitors **38**–**41.**

ASCT2 (also known as SLC1A5) is a major sodium-dependent transporter responsible for glutamine uptake [153], associated with the expression of oncogenic MYC and KRAS signaling and playing a role in many clinically relevant tumors [154,155]. Benzyloxy compound V-9302 (**38**, Chart 6, Table 2) significantly inhibited glutamine uptake mediated by the human ASCT2 isoform in HEK293 cells (IC_50_ = 9.6 μM) [153]. However, ASCT2 deletion in two human cancer cell lines did not abolish the cell growth inhibition by compound **38** or other ASCT2 inhibitors belonging to the same 2-amino-4-bis(aryloxybenzyl) aminobutanoic acid (AABA) class. AABA compounds inhibited glutamine transport even in ASCT2-deficient cells, but not in parental cells. Deletion of ASCT2 prompted the upregulation of SNAT2, another glutamine transporter, which was also inhibited by AABA compounds. Additionally, AABAs inhibited isoleucine uptake via LAT1, a transporter co-upregulated with ASCT2 in cancer cells [156], and essential for tumorigenesis in a KRAS-mutant model of CRC [157].

JPH203 (**39**) is anticancer drug targeting L-type amino acid transporter 1 (LAT1, also known as SLC7A5), which plays a primary role in the uptake of essential amino acids in tumor cells. It inhibits LAT1, with an IC_50_ value of 193 ± 50 nM. Compound **39** inhibits the uptake of branched-chain amino acids, including glutamine. Treatment of LAT1-positive HT-29 colon cancer cells with (**39**) resulted in a time-dependent reduction in leucine uptake activity. Its inhibitory effect on LAT1 was enhanced by preincubation, suggesting a synergistic mechanism when combined with co-incubation [158].

6-Diazo-5-oxo-norleucine (DON, **40**) is a glutamine antagonist originally isolated from the fermentation broth of a Streptomyces in the 1950s [159]. It inhibits several glutamine-utilizing enzymes by a two-step, mechanism-based inhibition [160,161]. Despite its broad enzymatic inhibition, compound **40** showed weak efficacy in vitro and poor tolerability in vivo [162]. JHU-083 (**41**) is a dual DON prodrug developed to overcome its limitations. Simple alkyl ester-based prodrugs of (**40**) failed due to the formation of diazoimine byproducts. A successful strategy involved masking both the amine and carboxylate groups. The resulting prodrugs exhibited rapid metabolism in mouse plasma, with several analogs showing excellent stability in monkey and human plasma. These prodrugs achieved superior tumor cell-to-plasma ratio compared to DON [163,164]. The prodrug (**41**) inhibited cell growth and induced apoptosis in human MYC-expressing medulloblastoma cell lines. It also sensitized C57BL/6 mouse cerebellar stem and progenitor cells in a MYC-driven medulloblastoma mouse model. In immune-competent animals bearing orthotopic tumors, treatment with compound **41** increased survival [165].

**Chart 7:**
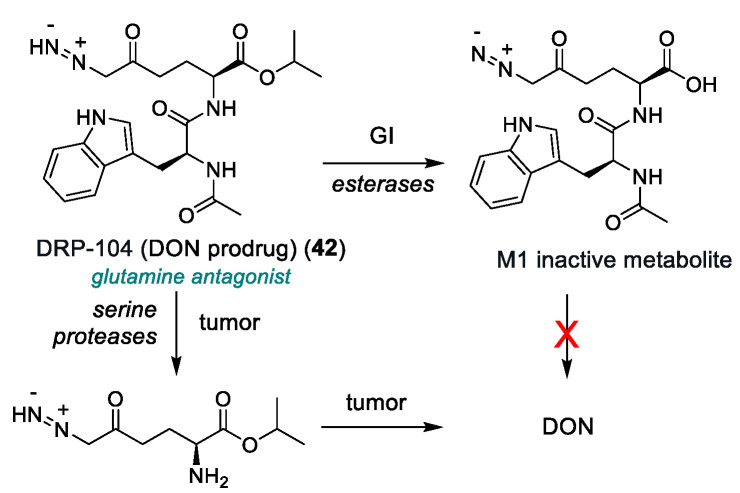
Chemical structures and bioactivation of **42.**

DRP-104, also known as Sirpiglenastat (**42**, Chart 7), is another DON prodrug that is selectively bioactivated to DON within tumor by serine proteases, while it is inactivated to the M1 metabolite in gastrointestinal tissue by carboxylesterases. This strategy improved drug delivery and reduced systemic toxicity. Compound **42** exhibited antitumor activity similar to (**40**), but with significantly lower toxicity. It reduced glutamine flux into the TCA cycle and affected multiple metabolic pathways in tumor, including those related to amino acids, nucleotides and carbohydrates/TCA cycle intermediates. The combination of compound **42** with anti-PD-1 immunotherapy showed superior efficacy compared to monotherapies. The antitumor activity of (**42**) depended on CD8+ T cells, resulting in sustained immunologic memory [166]. Additionally, compound **42** was shown to suppress KEAP1-mutant tumors frequently occurring in lung cancer by inhibiting glutamine-dependent nucleotide synthesis and promoting antitumor T cell responses [167].

**Chart 8:**
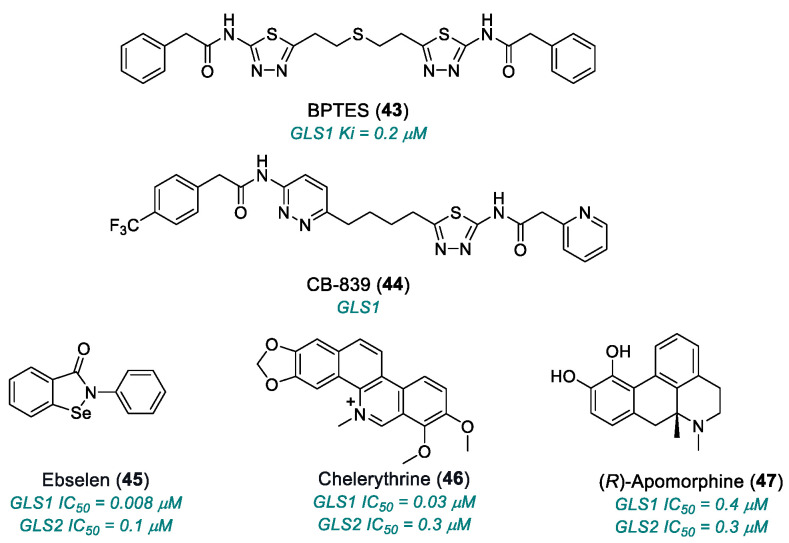
Chemical structures of glutamine metabolism inhibitors **43**–**47.**

BPTES (bis-2-(5-phenylacetamido-1,3,4-thiadiazol-2-yl)ethyl sulfide) (**43,** Chart 8) was shown to inhibit selectively the growth of glioma cells [168]. Kinetic analysis indicated that BPTES is a potent uncompetitive inhibitor of GLS1, with a Ki of 0.2 μM. Its structure differs significantly from that of glutamine. The kinetics of BPTES were characterized using human kidney-type glutaminase lacking the C-terminal sequence present in the KGA isoform, which is expressed in the kidney, brain, intestine and cells of the immune system, or in the GAC isoform, expressed in the heart, pancreas, placenta, lung and in many transformed cells [169]. Compound **43** was more potent than DON but exhibited poor solubility and low bioavailability. Attempts to improve these properties through structural analogs were unsuccessful [170,171]. Drug resistance to GLS1-specific inhibitors, such as compound **43** or its analogue, CB-839 (**44**), also known as Teleglenstat, may be due to the compensatory activity of GLS2, suggesting the need for dual GLS1/GLS2 inhibitors.

Screening of 1280 compounds from the LOPAC^1280^ library of pharmacologically active compounds led to the identification of ebselen (**45**), chelerythrine chloride (**46**) and (*R*)-apomorphine hydrochloride (**47**) as potent glutaminase inhibitors. All compounds showed time- and concentration-dependent inhibition of glutaminase activity. Compounds **45** and **46** were 5- to 10-fold, respectively, more potent against GLS1 than GLS2, with IC_50_ values of 0.008 μM and 0.1 μM for **45** (GLS1 vs GLS2), and IC_50_ = 0.03 μM and 0.3 for **46** (GLS1 vs GLS2), while compound **47** showed similar activity against both isoforms (IC_50_ = 0.4 μM and 0.3 μM). Mechanistic studies indicated non-competitive inhibition for (**45**) (possibly through covalent adduct formation) and competitive inhibition for (**46**) and (**47**) [162]. The uncompetitive inhibition by compound **45** aligns with previous results [172], suggesting the formation of a selenenylsulfide (–Se–S–) bond [172] with cysteine residues in the active site of glutaminase [173]. Both (**45**) and (**46**) may stabilize the enzyme in an inactive tetrameric configuration or disrupt tetramerization [162].

**Table 2:** Compounds targeting glutamine metabolism.

Name	Target	Year	Stage	Limitation	References
V-9302 (**38**)	ASCT2	2016	Preclinical studies	Effective only at micromolar range	[153,154,155,156,157]
JPH203 (**39**)	LAT1	2018	Phase I completed	Hepatotoxicity; limited PK data	[158]
DON (**40**)	Glutamine antagonist	1979	Phase I/II completed	Weak in vitro efficacy; poo3,172–172r in vivo tolerability; severe GI toxicity	[159,160,161,162]
JHU-083 (**41**)	Glutamine antagonist (DON prodrug)	2016	Phase I/II	Toxicity at high doses	[163,164,165]
DRP-104 (**42**)	Glutamine antagonist (DON prodrug)	2022	Phase I/II	Residual toxicity (GI and systemic); narrow therapeutic window; poor PK data	[166,167]
BPTES (**43**)	GLS1	2010	Preclinical studies	Limited solubility and stability; micromolar activity	[168,169,170,171]
CB-839 (telaglenastat) (**44**)	GLS1	2014	Phase I completed	Non-universal efficacy; moderate toxicity	[170,171]
ebselen (**45**)	GLS1	2015	Preclinical studies	Multi-target, redox reactive; off-target risk and mitochondrial toxicity	[162,172,173,174]
chelerythrine chloride (**46**)	GLS1	2015	Preclinical studies	Significant inhibition but with off-target cytotoxic effects	[162,172,173,174]
(*R*)-apomorphine hydrochloride (**47**)	GLS1, GLS2	2015	Preclinical studies	Low selectivity	[162,172,173,174]

### 2.3. Fatty Acid Synthesis

Lipid metabolism has attracted significant interest in cancer therapy because lipids are involved in many biochemical processes occurring in cancer initiation and progression [15]. In fact, lipids which comprise a variety of biomolecules made up of fatty acids, play key roles in cancer cell growth, homeostasis, energy metabolism and as structural components of cell membrane [174]. Moreover, they are involved in signal transduction cascades and can be converted into mediators of cancer development [175]. During cancer cell proliferation, the rapid formation of biological membranes demands increased energy and a high rate of lipid synthesis [14].

**Figure 7:**
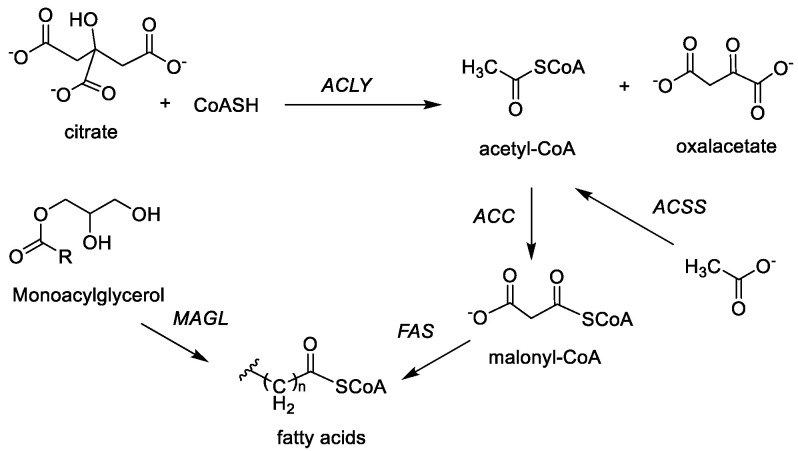
Fatty acid metabolism. ACC: acetyl-CoA carboxylase; ACLY: acetyl-CoA lyase; ACSS: acetyl-CoA synthetase short chain family member; FAS: fatty acid synthase; MAGL: monoacylglycerol lipase.

MAGL. Monoacylglycerol lipase (MAGL) oversees the conversion of monoacylglycerols into free fatty acids during lipogenesis in cancer cells, often due to mutation in metabolic pathways. The MAGL overactivation in tumor calls correlates with increased aggressiveness [176]. MAGL inhibition was shown to exert antitumor effects in several cancer cell lines [16,177]; however, the inhibition of MAGL may also cause neurodegenerative, inflammatory and metabolic side effects [178] (Figure 7).

**Chart 9:**
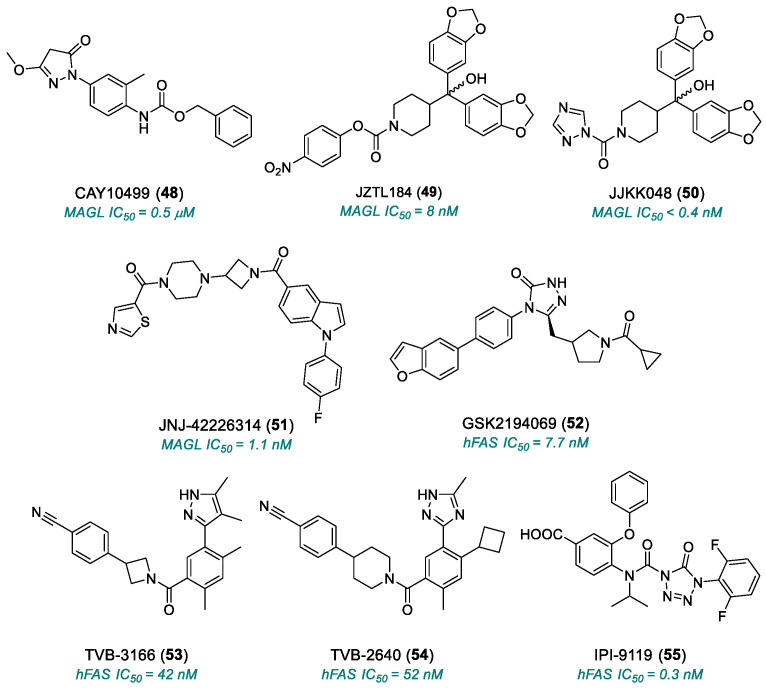
Chemical structures of MAGL inhibitors (**48**–**52**) and FAS inhibitors (**53**–**55**).

CAY10499 (**48**, Chart 9, Table 3) inhibited MAGL-mediated 4-nitrophenylacetate (4-NPA) hydrolysis, with IC_50_ values of 0.5 ± 0.03 μM and of 0.4 ± 0.04 μM using 2-oleoylglycerol (2-OG) as a substrate. Preincubation with compound **48** strongly increased the inhibition to nanomolar IC_50_ values, suggesting that it binds irreversibly to MAGL, even in the absence of a substrate [179]. Compound **48** was tested against several lipases, including adipose triglyceride lipase (ATGL), hormone-sensitive lipase (HSL), *sn*-1-diacylglycerol lipase (DAGL), monoacylglycerol lipase, a/b-hydrolase domain 6 and carboxylesterase 1 (CES1), using recombinant human and mouse enzymes, either in cell extracts or using purified enzymes. While (**48**) inhibited both mouse ATGL and HSL, it showed weaker inhibition of human HSL [180].

JZL184 (**49**), identified through activity-based protein profiling (ABPP) methods [181], is a potent and selective MAGL inhibitor (IC_50_ = 8 nM) that provides rapid and sustained MAGL blockade. In the mouse brain proteome, compound **49** potently and selectively inactivated MAGL and increased the 2-arachidonoylglycerol level (2-AG) in male C57Bl/6 mice [182]. It has been proposed for use in treating bone diseases associated with primary bone cancer and bone metastasis; however, activation of the skeletal endocannabinoid system may limit its osteoprotective potential [183]. In osteoblasts, treatment with (**49**) in the presence of multiple myeloma (MM) cell-derived factors reduced osteoblast proliferation, though not their maturation or bone nodule formation. In an in vivo assay, compound **49** caused moderate but significant bone loss in the long bones of immunocompetent mice inoculated with 5TGM1 MM cells [184].

JJKK-048 (**50**) was discovered by combining the bulky benzhydryl group of (**49**), required for selectivity, with a triazole leaving group, leading to a potent human and rodent MAGL inhibitor, with IC_50_ < 0.4 nM. It likely targets the catalytic serine residue (S122) of MAGL, forming an irreversible covalent bond. In HEK293 cells transiently overexpressing human MAGL (hMAGL), compound **50** inhibited 2-AG hydrolysis in a dose-dependent manner at nanomolar concentrations [185]. MAGL could promote hepatocellular carcinoma (HCC) progression via epithelial–mesenchymal transition (EMT), suggesting its role as a biomarker and potential therapeutic target [186]. Hypoxia-induced resistance to the multi-kinase inhibitor regorafenib, mediated by ABCG2 overexpression, was reduced by MAGL inhibition. Compound **50** lowered ABCG2 overexpression in MDA-MB-231 cells, enhanced regorafenib accumulation and improved its therapeutic response [187].

JNJ-42226314 (**51**) is a reversible and highly selective MAGL inhibitor (IC_50_ = 1.1 nM) via a competitive mechanism with respect to the 2-AG substrate. In in vivo studies, it produced antinociceptive effects in both inflammatory and neuropathic pain models. At 30 mg/kg, it induced hippocampal synaptic depression, altered sleep onset and decreased electroencephalogram gamma power. However, at 3 mg/kg, it still achieved approximately 80% enzyme occupancy, increased 2-AG and norepinephrine levels, and maintained antinociceptive activity without cognitive impairment [188].

FAS. Fatty acid synthase (FAS, FASN), encoded in humans by the *FASN* gene, is a key enzyme in the endogenous lipogenesis. It catalyzes the synthesis of palmitate from acetyl-CoA and malonyl-CoA using NADPH as a reducing agent [189].

GSK2194069 (**52**), developed at GSK, is a potent hFAS inhibitor, with an IC_50_ of 7.7 ± 4.1 nM (CoA-release assay) and 29 ± 3.2 nM (acetoacetyl-CoA assay) [190]. It showed little or no inhibition with b-hydroxybutyryl-CoA and crotonyl-CoA and did not inhibit the b-ketoacyl reductase (KR) domain. In gastric and non-small-cell lung cancer cell lines, compound **52** reduced de novo fatty acid synthesis. In the A549 non-small-cell lung cancer cell line, (**52**) also decreased phosphatidylcholine levels, with an EC_50_ of 15.5 ± 9 nM, correlating with reduced palmitate production. A crystal structure of compound **52** in the KR domain has been reported [191].

TVB-3166 (**53**) was reported as a FASN inhibitor (IC_50_ = 42 nM) with antitumor effects and potential clinical relevance. It showed inhibition in oral squamous cell carcinoma (OSCC) SCC-9 ZsG and metastatic LN-1A cells. Compound **53** significantly reduced cell viability and proliferation, promoted cell cycle arrest and apoptosis, increased adhesion to myogel in both OSCC cell lines and reduced cell migration [192].

TVB-2640 (**54**) is a selective, potent, reversible FASN inhibitor (IC_50_ = 52 nM) evaluated for non-alcoholic fatty liver disease (NAFLD) and non-alcoholic steatohepatitis (NASH) correlated with elevated hepatic de novo lipogenesis [193]. In the randomized clinical trial n. NCT03938246, ninety-nine patients received placebo or 25 mg or 50 mg of the drug (orally, once-daily for 12 weeks); compound **54** significantly reduced liver fat and improved biochemical, inflammatory and fibrotic biomarkers in a dose-dependent manner in patients with nonalcoholic steatohepatitis [194].

IPI-9119 (**55**) is an irreversible FASN inhibitor (IC_50_ of 0.3 nM) that acylates the thiesterase catalytic domain serine. It selectively inhibited the human purified FASN with negligible off-target effects and suppressed castration-resistant prostate cancer (CRPC) growth by reducing protein expression and transcriptional activity of both the full-length androgen receptor (AR) and the constitutively active AR variant V7 (AR-V7). In vivo, compound **55** inhibited AR-V7-driven CRPC xenografts and human metastatic CRPC-derived organoids and enhanced the efficacy of the androgen receptor inhibitor enzalutamide in CRPC cells [195].

**Chart 10:**
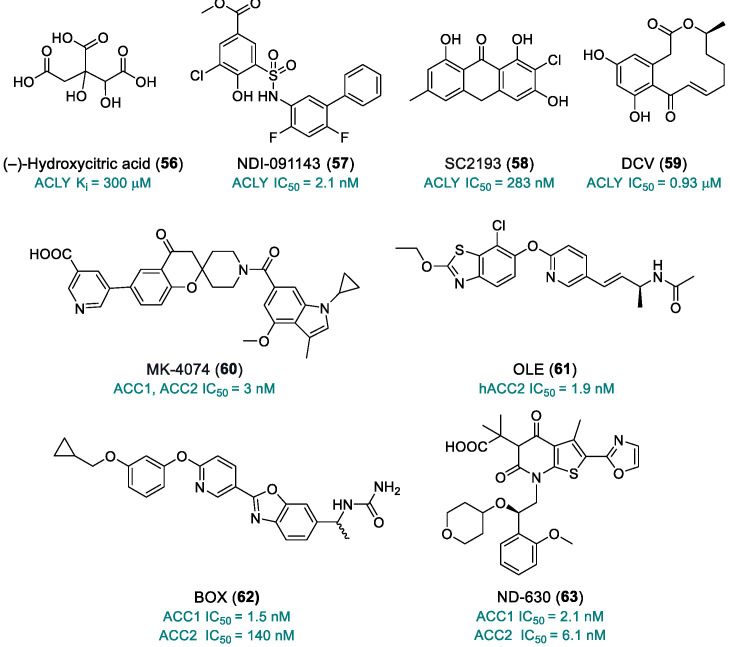
Chemical structures of ACLY (**56**–**59**) and ACC inhibitors (**60**–**63**).

ACLY. Human ATP citrate lyase (ACLY) is a cytosolic enzyme catalyzing the Mg-ATP-dependent conversion of citrate and CoA into acetyl-CoA and oxaloacetate [196]. In mammalian cells, it is mainly localized in the cytosol bound to endoplasmic reticulum, but was also found in the nucleus of human glioblastoma and colon carcinoma cells [197]. Given its central role in lipogenesis, ACLY is a promising target for the treatment of hyperlipidemia and hypercholesterolemia [198,199]. In cancer cells, ACLY plays a role between the glucose and lipid metabolism, two main metabolic alterations typical of tumors, being at the same time at the end of the glycolytic cascade and at the starting point of lipid synthesis. ACLY was shown to be a potential anticancer therapeutic target, since its inhibition interferes with both the glucose metabolism and lipid synthesis [198].

(–)-Hydroxycitric acid (**56**, Chart 10) is a naturally competitive ACLY inhibitor, with a Ki value of 300 μM. In mice implanted with syngeneic cancer cells, LL/2 Lewis lung carcinoma or MBT-2 murine bladder cancer cells, the triple combination (**56**) + lipoic acid + cisplatin or methotrexate showed enhanced antitumor efficacy [200].

NDI-091143 (**57**) was identified as part of a research project aimed at developing a class of 2-hydroxy-N-arylbenzensulfonamides as ACLY inhibitors. Compound **57** showed nanomolar inhibition of human ACLY, with IC_50_ values in the range of 2.1–4.8 nM, depending on the enzymatic assay, and proved to be competitive with the substrate, citrate [201,202,203]. Cryo-electron microscopy revealed an unexpected mechanism of inhibition: compound **57** binds to an allosteric hydrophobic pocket adjacent to the citrate-binding site, inducing extensive conformational changes in the enzyme that indirectly disrupt citrate binding [201].

SC2193 (**58**), 2-chloro-1, 3, 8-trihydroxy-6-methyl-9-anthrone, is another naturally occurring anthrone derivative extracted from a soil fungus. The compound showed an IC_50_ of 283 nM in the primary assay and, more specifically, a Ki of <100 nM against Mg citrate. It demonstrated specific and competitive inhibition of ACL with respect to the substrate Mg citrate and mixed non-competitive inhibition with respect to the essential cofactor Mg-ATP and CoA [204].

10,11-Dehydrocurvularin (DCV, **59**) is a fungal-derived natural macrolide lactone of dihydroxyphenylacetic acid, identified via chemical proteomics in living Jurkat cells (IC_50_ = 0.93 µM). It has demonstrated potential anti-infective, anticancer and immune-modulatory activities [205]. Compound **59** acts as an irreversible inhibitor of ACLY through its α,β-unsaturated carbonyl group, which serves as a Michael acceptor for the alkylation of cysteine-thiol groups in the protein. DCV showed strong antineoplastic activity by inhibiting acute T-lymphocyte leukemia Jurkat cells, as well as HCT116 colorectal carcinoma cells and HeLa cervical adenocarcinoma cells [206].

ACC. Acetyl-CoA carboxylase (ACC) is a biotin-dependent multidomain enzyme that catalyzes the conversion of acetyl-CoA to malonyl-CoA, a critical precursor for fatty acid synthesis via FAS [207]. Two main ACC isoforms have been reported in mammals: ACC1, which is primarily found in the cytosol of liver, adipose tissue and lactating mammary gland, and ACC2, which is commonly located in the outer mitochondrial membrane of oxidative tissues such as skeletal muscle, the heart and the metabolically active liver [14]. ACC1 was found to be upregulated in several types of cancer [208], while ACC2 functions as a key regulator of fatty acid β-oxidation [209].

MK-4074 (**60**) is a liver-specific, strong dual inhibitor of ACC1 and ACC2, with IC_50_ values around 3 nM. It effectively suppressed de novo lipogenesis (DNL) and enhanced hepatic fatty acid oxidation (FAO) in cultured hepatocytes, preclinical animal models and clinical studies. Its hepatoselectivity is attributed to its function as a substrate for organic anion transporting polypeptide (OATP) transporters, which are expressed exclusively in hepatocytes. In Phase 1 human studies, conducted in healthy individuals, multiple-dose administration of (**60**) (140 mg, 7 days) led to maximal DNL inhibition. In a separate short-term study evaluating hepatic steatosis, a 4-week treatment with compound **60** resulted in a 36% reduction in hepatic fat content, compared to an 18% reduction observed with pioglitazone [210].

OLE (**61**) was developed by replacing the alkyne unit with an olefin linker and modifying the aryl group at the central and left portions of Abbot compound A-908292. Compound **61** exhibited high hACC2 selectivity, with an IC_50_ of 1.9 nM, compared to an IC_50_ of 1950 nM for hACC1. These results were confirmed by in vivo studies, in which (**61**) was effective in C57BL/6 mice and showed no adverse cardiac effects or clinical signs in rats treated orally at 50 mg/kg/day for 4 days [211].

BOX (**62**) was discovered in a medicinal chemistry campaign aimed at reducing the undesirable body weight effects associated with a previous hit compound. Substitution of the acetamide group with an ureido moiety led to compound **62,** which exhibited improved selectivity for mouse ACC1 over ACC2, with IC_50_ values of 1.5 nM and 140 nM, respectively, and favorable PK in mice. Oral administration significantly reduced the malonyl-CoA concentration in HCT-116 xenograft tumors at doses exceeding 30 mg/kg. Furthermore, compound **62** showed significant antitumor efficacy in 786-O xenograft mice at an oral dose of 30 mg/kg [212].

ND-630 (**63**) is a potent allosteric ACC inhibitor discovered through structure-based drug design. It binds within the phosphopeptide acceptor and dimerization domain of ACC, thereby preventing dimerization and inhibiting the enzymatic activity of both ACC1 and ACC2 (IC_50_ = 2.1 nM and 6.1 nM). Compound **63** reduced fatty acid synthesis and promoted fatty acid oxidation in both cell cultures and animal models, displaying favorable drug-like properties. Chronic administration to diet-induced obese rats reduced hepatic steatosis, improved insulin sensitivity, decreased weight gain without affecting food intake and favorably affected dyslipidemia. In chronically treated Zucker diabetic fatty rats, (**63**) also reduced hepatic steatosis, enhanced glucose-stimulated insulin secretion and lowered hemoglobin A1c by 0.9% [213].

**Table 3:** Compounds targeting fatty acid synthesis.

Name	Target	Year	Stage	Limitation	References
CAY10499 (**48**)	MAGL	2008	Preclinical studies	Potential toxicity from normal lipid metabolism (liver, heart); poorly characterized bioavailability/toxicity; endocannabinoid accumulation	[180,181]
JZL184 (**49**)	MAGL	2009	Preclinical studies	Off-target effects at high doses; poorly characterized bioavailability/toxicity; endocannabinoid accumulation	[182,183,184]
JJKK-048 (**50**)	MAGL	2013	Preclinical studies	Off-target effects at high doses; poorly characterized bioavailability/toxicity; endocannabinoid accumulation	[186,187,188]
JNJ-42226314 (**51**)	MAGL	2020	Preclinical studies	Endocannabinoid accumulation	[189]
GSK2194069 (**52**)	FASN	2011	Preclinical studies	Limited oral bioavailability; metabolic and lipid side effects	[191,192]
TVB-3166 (**53**)	FASN	2015	Advanced preclinical studies	Metabolic and lipid side effects	[193]
TVB-2640 (**54**)	FASN	2017	Phase II	Risk of long-term resistance	[194,195]
IPI-9119 (**55**)	FASN	2020	Preclinical studies	Irreversible inhibitor	[196]
(–)-Hydroxycitric acid (**56**)	ACLY	2012	Preclinical studies	Low bioavailability	[201]
NDI-091143 (**57**)	ACLY	2019	Preclinical studies	No PK studies; limited toxicity data	[202,203,204]
SC2193 (**58**)	ACLY	2017	Preclinical studies	No PK studies; limited toxicity data	[205]
10,11-Dehydrocurvularin, DCV (**59**)	ACLY	2015	Preclinical studies	Poorly defined toxicity; instability; multi-target activity	[206,207]
MK-4074 (**60**)	ACC1, ACC2	2017	Preclinical studies	No PK studies; limited toxicity data	[211]
OLE (**61**)	hACC2	2018	Preclinical studies	No PK studies; limited toxicity data	[212]
BOX (**62**)	ACC1	2019	Preclinical studies	No PK studies; limited toxicity data	[213]
ND-630 (**63**)	ACC1, ACC2	2016	Preclinical studies	No PK studies; limited toxicity data	[214]

### 2.4. Structural Considerations of Metabolic Targets

Recent structural studies of key metabolic enzymes targeted by small-molecule inhibitors have provided crucial insights into the binding and mechanisms of these potential antitumor compounds. These enzymes, often featuring conserved catalytic cores or allosteric regulatory domains, present well-defined pockets suitable for selective targeting.

For example, LDHA contains a highly conserved catalytic site, where residues such as Arg168, His192 and Asp165 play a critical role in pyruvate binding and NADH-mediated reduction to lactate. Co-crystal structures of LDHA-inhibitor complexes, such as TODP (**5**), highlight direct interactions with these residues, explaining the observed potency and selectivity [42].

Similarly, in HK2, both the glucose and ATP-binding pockets are critical for enzymatic function. The interaction with VDAC1 at the mitochondrial membrane allows coupling with ATP synthesis. Structural models (e.g., PDB ID: 2NZT) demonstrate how small molecules and G6P-mimicking inhibitors, such as compounds **28–30**, exploit the enzyme’s flexible domain architecture [78]. This structural flexibility, particularly in the G6P allosteric site, enables the rational design of compounds that either compete with endogenous ligands or prevent conformational transitions required for activity.

GLUT1, a transmembrane transporter, has a central channel composed of helical bundles that accommodate glucose transport. Inhibitors such as STF-31 (**12**) and BAY-876 (**18**) bind within this channel, occluding glucose passage. Docking studies of GLUT1 analogs have highlighted roles for residues such as Phe 379 and Trp 388 in ligand binding [214].

Moreover, PKM2 represents another well-studied target, featuring both an active site and a regulatory allosteric pocket. The glycolytic flux can be modulated by allosteric activators (ML-265, **33**) or inhibitors such as (**31**), (**32**) or natural compounds (silibinin **34** and curcumin **36**), which can stabilize either the active tetrameric or inactive dimeric forms. Residues such as Arg399 have been implicated in conformational switching, particularly in the transition between inactive and active states, and are frequently involved in ligand binding [215].

Regarding FASN, crystal structures have resolved several domains, including the β-ketoacyl synthase and thioesterase domains, which are responsible for palmitate synthesis. Compounds such as TVB-2640 (**54**) bind selectively to the β-ketoacyl reductase domain, interacting with key catalytic residues, thereby disrupting lipid biosynthesis [216].

These structural insights guide the rational design of potent, selective inhibitors by targeting specific enzyme conformations or domains to overcome drug resistance and improve therapy. They also explain variations in compound efficacy across isoforms and tumor types, while the distinction between orthosteric and allosteric inhibition highlights the versatility of these targets and the opportunities to fine-tune therapeutic responses through domain-specific modulation.

## 3. Perspectives and Emerging Strategies in Tumor Metabolism

### 3.1. Metabolic Crosstalk and Compensatory Mechanisms

Numerous recent studies have highlighted how altered metabolic pathways in cancer do not operate in isolation but are highly interconnected and subject to functional compensation. This dynamic network, commonly referred to as *metabolic crosstalk*, presents a significant challenge to the efficacy of targeted metabolic therapies, yet also opens new avenues for more rational combinatorial strategies.

In particular, the inhibition of a single metabolic pathway often induces the compensatory activation of alternative routes. For example, glutaminase (GLS) inhibition has been shown to result in increased utilization of glucose and fatty acids, which can be metabolized through glycolysis or β-oxidation to support ATP production and the synthesis of biosynthetic intermediates. Furthermore, in certain cancer contexts, blockade of glutaminolysis has been associated with the reactivation of mitochondrial oxidative phosphorilation (OXPHOS), contributing to therapeutic resistance [217].

A promising strategy to overcome such compensations is the use of combination therapies. One notable example is AZD3965 (**11**), an inhibitor of the lactate transporter MCT1, which has been shown to enhance the activity of glutaminase inhibitors. This synergistic effect stems from the simultaneous inhibition of lactate efflux and glutamine metabolism, leading to redox collapse and suppression of tumor proliferation [218].

Similarly, inhibition of LDH-A, a key glycolysis enzyme, can be circumvented by cancer cells via enhancement of OXPHOS. In preclinical models of pancreatic cancer, treatment with GNE-140 (**3**), a potent LDH-A inhibitor, resulted in mitochondrial compensation, mediated by the activation of the AMPK-mTOR-S6K pathway. The combination of GNE-140 with phenformin (**4**), an OXPHOS inhibitor, successfully overcame metabolic resistance and restored treatment sensitivity [219].

The accumulation of metabolic intermediates from compensatory pathways can also contribute to the epigenetic modification of the tumor genome, with direct effects on gene expression and cancer progression. For instance, intracellular lactate levels can influence histone lactylation (HKla), an epigenetic mark recently associated with the regulation of pro-inflammatory and pro-tumor genes [220]. Thus, modulating metabolic crosstalk may also have significant epigenetic implications.

Overall, the expanding knowledge of tumor metabolic networks requires a paradigm shift: from single-target interventions to multi-target strategies capable of simultaneously inhibiting critical metabolic nodes and adaptive pathways. The identification and characterization of these adaptive circuits are increasingly facilitated by integrated metabolomics techniques, CRISPR-based screening systems and network modeling.

### 3.2. Emerging Technologies: PROTACs and Targeted Degradation of Metabolic Proteins

An area of growing interest in anticancer therapy is targeted protein degradation through technologies such as PROTACs (PROteolysis Targeting Chimeras). These bifunctional molecules promote the selective degradation of target proteins by recruiting specific E3 ligases, leading to polyubiquitination and subsequent proteasomal degradation.

Compared to traditional inhibitors, PROTACs offer several advantages: they act catalytically, require lower concentrations and can target previously considered “undruggable” proteins. Furthermore, by eliminating the entire protein rather than merely inhibiting its active site, PROTACs reduce the likelihood of resistance driven by binding site mutations [221].

Recent studies have reported promising preclinical results for PROTACs targeting metabolic enzymes such as LDH-A, PKM2 and GLS1. Some of these agents have been designed as tumor-selective pro-drugs, activated under specific enzymatic or acidic conditions present in the tumor microenvironment, thus improving selectivity for neoplastic cells [222].

Other emerging technologies include AUTACs (Autophagy-Targeting Chimeras) and ATTECs (Autophagosome-Tethering Compounds), which exploit the autophagic pathway to degrade not only proteins but also organelles and metabolic aggregates. These approaches offer novel therapeutic possibilities, particularly in tumors reliant on organelles such as mitochondria or lipid droplets [223].

### 3.3. Precision Metabolic Oncology: New Therapeutic Frontiers

The marked metabolic heterogeneity both between patients and within individual tumors presents a challenge, but also a unique opportunity, for personalized therapy. The integration of high-resolution metabolomics, multi-omics profiling and technologies such as single-cell RNA sequencing enables the identification of tumor-specific metabolic signatures and the prediction of drug responses.

A prominent example is DRP-104 (**42**), a prodrug derived from DON (**40**), which is selectively activated in the tumor microenvironment. Compound **42** has demonstrated not only direct antitumor effects but also the ability to enhance antitumor immunity, acting synergistically with anti-PD-1 antibodies in mouse models [224].

These findings suggest that metabolic modulation not only affects the intrinsic characteristics of tumor cells but can also reshape the tumor microenvironment, providing valuable support for immunotherapeutic strategies.

### 3.4. Concluding Remark

The identification of redundant metabolic pathways and crosstalk circuits, coupled with the advent of emerging technologies, such as PROTACs and targeted degradation platforms, is revolutionizing the landscape of metabolic cancer therapy. The future of the field lies in the integration of rational combinatorial approaches, grounded in a robust understanding of adaptive mechanisms and systems biology. In this context, precision metabolic medicine is no longer a distant goal, but a tangible and achievable reality.

## 4. Summary and Conclusions

Over the past decades, cancer metabolism has emerged as a promising and rapidly expanding field for the development of novel anticancer strategies. The reprogramming of metabolic pathways, long recognized as a hallmark of cancer, provides malignant cells with the necessary biochemical resources to support uncontrolled proliferation, resistance to apoptosis, immune evasion and metastasis. This review has highlighted recent advances in the identification and characterization of small-molecule inhibitors targeting key metabolic enzymes involved in glycolysis, glutaminolysis and fatty acid synthesis—processes that are consistently upregulated in various tumor types.

By targeting enzymes such as LDHA, GLUT1, HK2, PKM2, GLS1/2, FASN, ACLY and ACC, researchers have demonstrated the feasibility of disrupting tumor bioenergetics and biosynthetic machinery. Notably, several of these compounds, including BAY-876 (**18**), ML-265 (**33**), TVB-2640 (**54**) and DRP-104 (**42**), have progressed to preclinical or clinical stages, underscoring the translational potential of metabolism-targeting therapies. Additionally, the structural and mechanistic diversity of these inhibitors, including orthosteric, allosteric, covalent and substrate-mimicking strategies, offers new avenues for specificity and reduced off-target effects.

Moreover, the therapeutic relevance of metabolic modulation extends beyond tumor cell-intrinsic effects. Compounds such as (**42**) and MAGL inhibitors have demonstrated the ability to reshape the tumor microenvironment and potentiate antitumor immune responses. This aligns with a broader shift toward combinatorial approaches that integrate metabolic inhibitors with immunotherapies, chemotherapeutic agents, or targeted drugs.

Nevertheless, several challenges remain. Tumor heterogeneity, metabolic plasticity, and compensatory pathways can undermine the efficacy of single-agent treatments. The development of reliable metabolic biomarkers, patient stratification strategies and predictive models of response will be crucial to guide clinical application. Furthermore, a deeper understanding of the systemic effects and potential toxicities associated with long-term metabolic inhibition is needed.

Recent insights into metabolic crosstalk and compensatory mechanisms have further highlighted the need for multi-targeted and context-specific approaches. The emergence of targeted protein degradation technologies, such as PROTACs, and advances in metabolomic profiling are paving the way for precision oncology strategies that exploit tumor-specific metabolic vulnerabilities.

In conclusion, targeting tumor metabolism represents a compelling and versatile approach in the anticancer drug discovery landscape. Continued interdisciplinary efforts combining medicinal chemistry, structural biology, cancer biology and translational research will be key to unlocking the full therapeutic potential of metabolic interventions and delivering innovative treatments to patients with high unmet medical needs.

## Abbreviations

The following abbreviations are used in this manuscript:

AAT	aspartate aminotransferase
ACC	acetyl-coa carboxylase
ACLY	human atp citrate lyase
AIF	apoptosis-inducing factor
ASCT2	alanine, serine, cysteine transporter 2
ATP	adenosine triphosphate
BBB	blood–brain barrier
CDKs	cyclin-dependent kinases
CRPC	castration-resistant prostate cancer
DLBCL	diffuse large b cell lymphoma
DNMT	DNA methyltransferase
ECM	extracellular matrix
FADH	flavin adenine dinucleotide
FAS	fatty acid synthase
FASN	fatty acid synthase
G6P	glucose-6-phosphate
GLS1	glutaminase 1
GLUT	glucose transporter
GLUT1	glucose transporter type 1
GLUT1DS	glucose transporter type 1 deficiency syndrome
GOT	glutamic-oxaloacetic transaminase
GPT	glutamic–pyruvic transaminase
HCC	hepatocellular carcinoma
HK	hexokinase
HKla	histone lysine lactylation
HNSCC	head and neck squamous cell carcinoma
LAT1	l-type amino acid transporter 1
LDH	lactate dehydrogenase
LDHA	lactate dehydrogenase type a
LDHB	lactate dehydrogenase type b
MAGL	monoacylglycerol lipase
MCT1	monocarboxylate transporter 1
NADH	nicotinamide adenine dinucleotide
NHL	non-Hodgkin lymphomas
NSCLC	non-small-cell lung cancer
OMM	outer mitochondrial membrane
OXPHOS	oxidative phosphorylation
PK	pyruvate kinase
PKM1	pyruvate kinase m1
PKM2	pyruvate kinase m2
RCCs	renal cell carcinomas
ROS	reactive oxygen species
SAR	structure-activity relationship
TCA	tricarboxylic acid
TMJOA	temporomandibular joint osteoarthritis
TNBC	in triple-negative breast cancer
VDAC	voltage-dependent anion channels
VHL	von Hippel–Lindau
